# Effect of supplementing hydroxy trace minerals (Cu, Zn, and Mn) on egg quality and performance of laying hens under tropical conditions

**DOI:** 10.5713/ab.22.0416

**Published:** 2023-06-26

**Authors:** Vasan Palanisamy, Sakthivel PC, Lane Pineda, Yanming Han

**Affiliations:** 1 Department of Animal Nutrition, Veterinary College and Research Institute, Namakkal, Tamil Nadu Veterinary and Animal Sciences University, Tamil Nadu 637002, India; 2 Trouw Nutrition R&D, 3811, MH Amersfoort, The Netherlands

**Keywords:** Copper, Egg Production, Egg Quality, Laying Hen, Manganese, Trace Minerals, Zinc

## Abstract

**Objective:**

A pivotal study was designed to investigate the effect of Hydroxy (HYC) Cu, Zn, and Mn on egg quality and laying performance of chickens under tropical conditions.

**Methods:**

A total of 1,260 Babcock White laying hens (20-wk-old) were randomly assigned to one of 4 treatments with 15 replicates of 21 hens each in a Randomized Complete Block Design. The birds were reared for 16 weeks and were fed the corn-soybean meal diets supplemented with one of the following mineral treatments: T1, inorganic (INO, 15 ppm CuSO4, 80ppm MnSO4 and 80 ppm ZnO); T2, Hydroxy-nutritional level (HYC-Nut, 15 ppm Cu, 80 ppm Mn, 80 ppm Zn from Hydroxy); T3, Hydroxy-Low (HYC-Low, 15 ppm Cu, 60 ppm Mn, 60 ppm Zn from Hydroxy); T4, Hydroxy plus inorganic (HYC+INO, 7.5 ppm HYC Cu+7.5 ppm CuSO4, 40 ppm HYC ZnO+40 ppm ZnSO4, 40 ppm HYC Mn+ 40 ppm MnSO4). The egg production was recorded daily, while the feed consumption, feed conversion ratio (FCR) and egg mass were determined at the end of each laying period. The egg quality parameters were assayed in eggs collected over 48 h in each laying period.

**Results:**

Overall, no significant effect of treatments was observed on percent egg production, egg weight and FCR (p>0.05). Feed intake was significantly lower in birds fed Hydroxy plus inorganic (p<0.05) diet. The supplementation of HYC-Low significantly increased the egg mass compared to the other treatments (p<0.05). HYC supplementation alone or in combination with INO elicited a positive effect on shell thickness, shell weight, shell weight per unit surface area, yolk colour, albumen and yolk index for a certain period (p<0.05), but not throughout the whole laying period.

**Conclusion:**

Dietary supplementation of HYC-Low (15-60-60 mg/kg) showed similar effects on production performance and egg quality characteristics in laying hens as compared to 15-80-80 mg/kg of Cu-Zn-Mn from inorganic sources. This indicates that sulphate based inorganic trace minerals can effectively be substituted by lower concentration of hydroxyl minerals.

## INTRODUCTION

Trace minerals (TM) are essential in the diets of laying hens and play a specific key role in the pathways of shell formation and egg production. Zinc (Zn) is a cofactor of carbonic anhydrase, which is essential for shell deposition [1]. Manganese (Mn) is an activator of enzymes involved in the synthesis of glycosaminoglycans and glycoproteins, which are important in the formation of the organic matrix of the shell [2]. Copper (Cu) is an integral part of the lysyl oxidase that catalyzes the cross linking of collagen and elastin in the eggshell membrane [3].

As an industry practice, the diets of laying hens are typically supplied with TM in the form of inorganic compounds, i.e. oxides, sulfates, etc. However, inorganic (ITM) source interacts with dietary components forming insoluble complexes and reduces mineral availability [4]. Consequently, ITM are usually provided more than the recommended amount to cover basic requirement and prevent deficiency. However, dietary minerals more than the requirements are excreted in the litter contributing to environmental pollution [4].

Hydroxy TM (HTM) is the newest and most technologically advanced form of TM with high bioavailability and low environmental impact. These TM are characterized by unique crystalline structure and low solubility, which promotes better stability in feed, improved bioavailability, and increased efficacy. In contrast with oxide/sulphate inorganic sources, HTM do not go into solution nor disassociate early in the digestive tract resulting in more Cu, Zn and Mn being delivered for absorption and utilization in the lower intestinal tract [5].

Recently, several animal trials have focused on the supplementation of this new generation TM. The result of a trial in laying hens in which different sources and dosages of TM (HTM vs ITM) were evaluated showed that HTM decreased the percentage of cracked eggs, increased egg production and improved efficiency of laying [6].

The objective of this study was to validate the efficacy of HTM under Indian tropical condition and evaluate its effect on egg quality and laying performance of chickens.

## MATERIALS AND METHODS

### Experimental birds and diets

The present layer chicken trial and procedures were reviewed and approved by Institutional Animal Ethical Care committee of Tamil Nadu Veterinary and Animal Sciences University (13/VCRI-NKL/2021, dt;26.10.2021).

One thousand two hundred and sixty (1,260) 20-wk-old laying hens (Babcock White) were purchased from a commercial company and randomly allocated in a randomized complete block design to four treatments with fifteen replicates of 21 hens each. A four-week period was used to adapt the hens to the experimental diets, followed by an experimental period of 16 weeks (4 periods of 28-d laying cycles each). Hens were placed in battery cages (three hens per cage) and reared in an open-sided housing. Hens were fed ad libitum according to their assigned treatment and they always had free access to water from the start till the termination of the trial. They were provided with 16 h of light and 8 h of darkness per day.

A basal mineral premix free from Cu, Zn and Mn was formulated and prepared prior to the preparation of the treatment diets. Supplemental TM was added according to the treatments (Table 1) on top of the basal premix and manually mixed before its incorporation in the basal diet. A practical corn-soybean meal, AGP-free layer diet was formulated to meet or exceed nutrient recommendations for Babcock White layer chicken (Table 2).

### Production performance

The measurements were performed for four periods and each period consisted of 4 weeks. Egg production was recorded daily, whereas feed consumption was determined every 28 days throughout the experiment. At the end of each laying period, total egg production and percent egg production were calculated for each experimental unit. Eggs collected over a 48-hour period were saved for egg grading and weight determination of every period. Feed conversion ratio was calculated as grams of egg mass produced per gram of feed consumed. Saleable eggs were calculated at the end of each period.

### Egg quality parameters

During each laying phase, about 100 eggs were collected randomly from each treatment (representing fifteen replicates) on 27th and 28th day egg collection. Eggs collected over 48-h in each period (n = 400) were used to evaluate the egg quality parameters viz., eggshell defects (cracked, soft shelled), eggshell thickness, eggshell weight and shell weight per unit surface area (SWUSA). Eggshell thickness without membrane was measured at three places (equatorial region, broad and narrow ends) by using a digital screw gauge with 0.01 mm accuracy and averaged.

SWUSA: The surface area of egg was calculated first by using the following formula:


(12.6 [(Length+Width)/4]2)/100

Then, SWUSA was calculated by using the formula:


=shell weight/surface area.

### Statistical analysis

Data were tested for normality and analysed using the general linear model procedure in SAS appropriate for RCBD. Significant differences between treatment groups were detected by Tukey kramer honestly significant difference test. The differences among the treatment were considered significant at p-value (<0.05).

## RESULTS

The effect of treatments on production performance of White Leghorn layer chicken during four laying periods and overall experimental period (25 to 40 wks) is presented in Table 3.

### Production performance

The supplementation of HTM minerals did not significantly affect the egg production during the first two and fourth periods of laying. However, in the third laying period, the egg production was significantly (p<0.05) reduced in hens fed with HYC+INO diets compared to the rest of the treatments (Table 3). Throughout the study period, the daily feed intake was significantly lower in the HYC+INO group (p<0.05). The ADFI of the other treatments was comparable in the whole period of laying (p>0.05). The FCR of HYC+INO mineral fed birds was better compared to INO mineral supplemented hens during the first period of laying. However, the difference disappeared in the succeeding period and no difference was observed among the treatments (p>0.05). No significant effect of treatment was observed on egg mass during the first three periods of laying. However, in the last laying phase, a marked reduction in egg mass was observed with the supplementation of HYC+INO (p<0.05). The dietary treatments did not affect the egg weight during the whole experimental period (p>0.05).

Overall, the results showed that the supplementation of HYC either alone or in combination with INO did not elicit significant improvement on percentage egg production, egg weight and FCR (p>0.05). The feeding of a diet with HYC+ INO significantly reduced the ADFI compared to the rest of the treatments (p<0.05). The supplementation of HYC-Low has a profoundly significant effect on egg mass (p<0.05).

### Egg quality

The effect of copper, zinc, and manganese hydroxychloride supplementation on egg quality parameters during four laying periods are presented in Table 4.

Dietary supplementation of different mineral sources did not have any significant effect (p>0.05) on shell thickness during first, third and fourth laying phases. However, during second phase the HYC+INO mineral supplemented group recorded thicker (p<0.001) shells. Neither INO mineral supplementation nor HTM supplementation at doses similar to INO had any significant difference (p>0.05) in shell weight during first laying period. On the other hand, HYC-Low and HYC+INO fed groups showed significantly higher (p<0.001) shell weight. During third laying phase, marked reduction (p<0.001) in shell weight was observed in INO group when compared to other treatment groups.

The mean shell weight per unit surface area (SWUSA) was markedly lower in INO group during first laying phase and HYC-Low group during third laying phase respectively. No significant impact (p>0.05) of mineral sources on percent broken and saleable eggs was observed during all the four laying periods.

## DISCUSSION

The role of TM in a variety of physiological processes that are essential for optimal bird growth, development and egg production are well documented [7]. Certain minerals viz., manganese and zinc function as enzymes cofactors involved in the synthesis of mucopolysaccharides and carbonate, which are essential for eggshell formation and quality [8]. These micronutrients cannot be stored in the body; therefore, regular dietary intake is required to meet functionality requirements [9]. Traditionally, the major sources of mineral supplements for poultry have been its inorganic salts viz., copper sulphate, manganese sulphate and zinc oxide/zinc sulphate. Recently, focus of mineral nutrition has been shifted towards the role of hydrolyzed inorganic metal complexes in laying hens and broilers.

The objective of this research was to investigate the effects of HYC Cu, Zn and Mn on laying performance and egg quality under tropical conditions. In the present study either complete or partial substitution of inorganic mineral sources with HYC mineral source had no effect on percent egg production, egg weight and egg mass during first two laying periods. However, partial (50%) substitution of INO sources with HTM markedly (p<0.05) decreased percent egg production and egg mass during fourth laying period. On the other hand, HTM led to marked reduction in ADFI during all the four laying phases. The speculative mechanism behind reduced ADFI might be lesser bioavailability of TM due to antagonistic effect of INO and HTM which should be investigated further. No difference was observed in the feed conversion ratio throughout the experimental period except for first laying phase during which better FCR was evidenced in HYC+INO supplemented group.

In contrast to the present study, it was reported that supplementation of different sources of copper (inorganic or organic Cu) did not exert any significant effect on nutrient metabolism of broilers [10], which could be due to lower Cu absorption as it was provided as a feed additive. On the contrary, it was reported that chicken treated with copper oxide utilise feed better than those supplemented with CuSO4 and Cu acetate [11] which was attributed to higher relative bioavailability of copper. It is possible that increased Cu levels might enhance the activities of the enzymes involved in nutrient utilisation [12,13].

Similarly, the present findings were in contrast to the findings of Tabatabaie et al [14] who observed that the source or level (25 and 50 ppm) of zinc supplements did not affect feed intake or feed conversion ratio in laying hens. However, highly significant impact was observed in feed intake, egg production, egg weight and egg mass of birds (22 to 34 wks of age) fed with the highest level (100 mg/kg) of zinc Methionine [15]. This was also supported by another study in 52 weeks old laying hens fed diets supplemented with Zn-Met vs ZnO nanoparticles that led to greater egg production rate, egg weight, and egg mass [16]. These results may be attributed to the high bioavailability of organic Zn. The second school of thought postulates that dietary Zn possibly improves egg production by interacting with the endocrine system. Zinc is essential for progesterone synthesis and its deficiency can induce an excessive secretion of prolactin hormone that initiates the broodiness and stops egg production [17].

Apart from Cu and Zn, it was also opined that manganese at different levels or from different sources (Mn-sulphate or Mn-AA) did not show any significant effect on egg production, egg weight, egg mass and feed conversion ratio in aged laying hens [18] and laying ducks [19]. Further, supplemental Mn (120 mg/kg feed) did not appear to have any effect on egg production, egg weight, egg mass or FCR in laying hens [20].

Even though all the three TM are involved in the nutrient metabolism, Zn is considered as the first limiting TM element among the three TM tested in this study and plays a key role in feed intake regulation [21,22]. The earliest observed effect of zinc deficiency was reduced feed intake by animals, which may be related to the role of zinc in inducing appetite [23]. Zinc functions mostly in enzyme systems involved in the metabolism of carbohydrates and lipids, in which it activates enzymes catalysing biochemical transformations, viz., pyruvate carboxylase, glycoside transferase, arginase, glutamine synthetase and superoxide dismutase. Reduced expression of zinc dependent-messenger RNA needed to synthesise these enzymes justifies the crucial role of zinc in carbohydrate metabolism [24]. Hence, in the present study, the lower ADFI observed in the HYC+INO supplemented group throughout the four laying phases might be attributed to zinc bioavailability which may not be enough to elicit normal feed intake. Under such sub-optimal conditions, Zn mainly affects bird’s daily feed intake, thereby leading to deficiency of primary nutrients that was reflected in marginal drop in percent egg production and egg mass throughout the experiment.

Combinations of minerals (Zn, Cu and Mn) irrespective of different sources (methionine hydroxyl analog vs sulphate diets) did not affect egg production, broken egg ratio, feed consumption or feed conversion ratio among treatments in 48 wks old laying hens [25]; 72 to 80 weeks old layers [26]; 37 weeks old laying hens [27] and 47 to 52 weeks old laying hens [28]. Similarly, different levels of dietary inorganic and organic Zn and Mn supplementation in 68 wks old laying hens showed no significant effect on egg production, egg mass and feed conversion ratio (p>0.05) among treatments [29]. In another study, combination of these three elements in organic form when supplemented at reduced levels as against inorganic sources did not significantly influence the egg production, egg weight and feed conversion ratio of 39 to 50 weeks-old laying hens [30]. This further confirmed that complete replacement of the inorganic form of Cu, Zn, Mg, and Fe by organic form of minerals maintained production performance at dosage 12 times lower as compared to the commercial inorganic level [31]. Supplementation of different amounts of TM combinations (organic or inorganic Zn, Mn, Cu, and Cr) in 50 weeks old laying hens did not lead to any significant differences in feed intake, feed conversion ratio and egg weight among experimental treatments [32]. On the contrary, supplementation of layer diet (38 to 53 weeks of age) with Zn, Mn, and Cu from the metal-amino acid complexes compared with oxide sources of the TM significantly increased (p<0.05) daily feed intake and feed conversion ratio, but simultaneously decreased percentage of broken eggs for the whole experimental period [33]. However, in the present study supplementation of layer diet (25 to 40 weeks of age) with HYC+INO combination significantly (p<0.05) reduced ADFI and egg mass for the whole experimental period. On the other hand, no significant differences were observed in percent egg production, egg weight and feed conversion ratio due to partial or complete substitution of INO minerals with HYC minerals.

It should be noted that basal diet composition, source of substituted inorganic minerals (oxide or sulphate), substitution rate of inorganic with hydroxy forms of the minerals are some of the main parameters involved in the obtained relatively inconsistent results.

Either partial or complete replacement of inorganic TM with hydroxy TM produced similar shell thickness throughout the study period at dosages 25 per cent lower Zn and Mn as compared to INO level. On the contrary, the highest values of shell thickness were achieved by birds (60 to 70 weeks of age) fed diets supplemented with Zn-Met at half the dose of inorganic ZnO as compared with control basal diet [34]. On the other hand, no significant difference in shell thickness and weight of egg components were observed in laying hens fed with different levels of organic Zn [35]. Similarly, different levels of manganese (Mn sulphate) supplementation did not evince any significant effect on eggshell thickness in laying ducks [19]. However Xiao et al [18] reported that different Mn levels (0, 25, 50, 100, or 200 mg/kg diet) or sources (Mn sulphate or Mn-amino acid) improved eggshell thickness significantly in laying hens. Mn activates alkaline phosphatase, which is a Zn containing enzyme that is involved in bone and eggshell calcification processes [36]. Zn plays a key role in formation of eggshell in the magnum and uterus during the deposition of albumen and isthmus in which membranes of eggshell are produced [34]. Zn plays a vital role in protein synthesis thereby enhancing the quality of the epithelium and eggshell membranes [37].

It was reported that laying hens (48 wks of age) fed with TM combinations in metal-amino acid (Cu-MHA, Zn-MHA, and Mn-MHA) form significantly increased shell thickness (p<0.05) as compared to sulphate forms [38]. On the contrary, it was recently reported that the dietary Fe, Cu, Mn, and Zn supplementation levels or different sources had positive effect (p>0.05) on eggshell thickness [39]. In the present study, the percentage of broken eggs were not influenced either by age of laying hens or by different combinations of TM sources. These findings agreed with another study [33] that also did not report any significant interaction of hen age and trace mineral sources on egg quality parameters. Supplementation of inorganic TM at higher levels when substituted with organic sources will not show any remarkable effects on egg quality traits. In contrast, supplementation of lower levels (40 mg/kg diet) of inorganic Zn and Mn significantly reduced eggshell thickness and increased the percentage of broken eggs as compared with same levels of (40;40;7 mg/kg of Zn, Mn, and Cu) metal-amino acid combinations [32]. In the present study also, the supplementation levels of inorganic mineral sources were high, because of which, substitution with HYC minerals had no marked effect on eggshell thickness and percent broken eggs. Partial substitution of INO TM with HYC minerals significantly increased the shell weight during first and third laying phases (Table 4) which may be attributed to higher retention efficiency of the minerals in the eggshell (REG). Even though, the retention efficiency values of the TM had not been assayed in the present study, related literatures revealed that REG of laying hens supplemented with organic forms of TM was significantly higher than those supplemented with inorganic sources [40–43,33].

## CONCLUSION

In conclusion the present investigation reveals that dietary supplementation of HYC-Low (15-60-60 mg/kg) showed similar effects on production performance and egg quality characteristics in laying hens as compared to 15-80-80 mg/kg of Cu-Zn-Mn from inorganic sources. This indicates that inorganic TM can effectively be substituted by lower concentration of hydroxy minerals.

## Figures and Tables

**Table 1:** Description of experimental treatments

Items	Nutritional level			

	Inorganic (INO)	HYC-Nut	HYC – Low	HYC+INO
CuSO_4_ (ppm)	15	-	-	7.5
ZnO (ppm)	80	-	-	40
MnSO_4_ (ppm)	80	-	-	40
HYC Cu (ppm)	-	15	15	7.5
HYC Zn (ppm)	-	80	60	40
HYC Mn (ppm)	-	80	60	40

HYC, hydroxychloride; INO, inorganic.

**Table 2:** Ingredient and nutrient composition of basal layer feed

Items	Value
Ingredient (g/kg)	
Maize	472.0
DORB	106.5
SFOC	100
Soya	216.0
Salt	3.4
Shell grit	65.0
Calcite	30.0
DCP	2.5
Methionine	1
Sodium bicarbonate	0.5
TM mix^1)^	1.0
Choline chloride (60%)	1
Vitamin premix^2)^	0.1
Vitamin premix^3)^	0.5
Livoliv (liv 52)	0.5
Total	1,000.0
Nutrient	
ME (kcal/kg)	2,470
CP (%)	17.79
CF (%)	6.76
Dig Lys (%)	0.79
Dig Met (%)	0.36
Ca (%)	3.58
AP (%)	0.28
Cu (ppm)	11.42
Zn (ppm)	40.35
Mn (ppm)	29.74

DORB, deoiled rice bran; SFOC, sunflower deoiled cake; DCP, dicalcium phosphate; ME, metabolizable energy; CP, crude protein; CF, crude fibre; AP, available phosphorus.

1) TM mix: each kg consists of FeSO_4_, 100 g; KI, 4 g; Na.Selenite, 1 g; COSO_4_, 5 g.

2) Vitamin premix: each gm consists of Vit.A, 82,500 IU; Vit.B_2_, 50 mg; Vit.D_3_, 12,000 IU; Vit.K, 10 mg.

3) Vitamin premix: each kg consists of Vit.B_1_, 8,000 mg; Vit.B_6_, 16,000 mg; Vit.B_12_, 8,000 mcg; Vit.E, 1,250 mg; Cal. C.Pantothenate, 5,000 mg; Niacin, 125 g;Ca, 125 g; Phosphates, 300 g.

**Table 3:** Effect of Inorganic and Hydroxy Cu, Zn and Mn on production performance at various periods of laying

Items	INO	HYC-Nut	HYC-Low	HYC+INO	Block	Treatment
25 – 28 weeks						
Egg production (%)	91.37	91.46	92.05	90.40	0.007	0.978
ADFI (g)	110.54^A^	108.06^B^	109.15^B^	104.12^C^	0.197	<0.0001
Egg weight (g)	53.88	54.48	53.90	53.81	0.88	0.728
FCR	2.250^a^	2.173^ab^	2.203^ab^	2.147^b^	0.00	0.04
Egg mass	49.24	49.83	49.63	48.66	0.01	0.53
29 – 32 weeks						
Egg production (%)	91.85	91.50	93.54	92.76	0.017	0.805
ADFI (g)	107.71^a^	106.07^b^	107.2^ab^	104.56^c^	0.278	<0.004
Egg weight (g)	53.84	54.61	54.50	54.15	0.55	0.119
FCR	2.182	2.126	2.101	2.085	0.07	0.121
Egg mass	49.45	49.98	50.98	50.23	0.035	0.474
33 – 36 weeks						
Egg production (%)	90.88	88.73	91.61	88.56	0.001	0.774
ADFI (g)	115.79^A^	113.46^B^	114.99^A^	109.33^C^	0.764	<0.0001
Egg weight (g)	55.99	56.18	56.16	55.98	0.25	0.29
FCR	2.278	2.281	2.238	2.214	0.004	0.544
Egg mass	50.88	49.86	51.45	49.58	0.0024	0.584
37 – 40 weeks						
Egg production (%)	88.33^a^	84.71^ab^	88.68^a^	81.67^b^	0.01	0.026
ADFI (g)	115.60^A^	113.80^A^	114.0^A^	109.50^B^	0.705	<0.003
Egg weight (g)	56.96	57.19	58.24	57.17	0.24	0.408
FCR	2.283	2.339	2.190	2.320	0.061	0.217
Egg mass	50.31^a^	48.43^ab^	51.63^a^	46.70^b^	0.17	0.030
25 – 40 weeks						
Egg production (%)	90.61	89.10	91.47	88.30	0.0005	0.261
ADFI (g)	112.20^A^	110.15^B^	110.96^AB^	106.37^C^	0.491	<0.0001
Egg weight (g)	55.17	55.62	55.70	55.28	0.35	0.258
FCR	2.248	2.229	2.183	2.192	0.002	0.201
Egg mass	49.97^ab^	49.52^b^	50.92^a^	48.79^c^	0.0054	0.031
Livability (%)	100	99.68	100	98.68	0.04	0.132

n = 15 replicates with 21 birds per replicate.

INO, inorganic; H, hydroxychloride; ADFI, average daily feed intake; FCR, feed conversion ratio;

a–c Means in a row bearing different superscripts are significantly different (p<0.05).

A–C Means in a row bearing different superscripts are significantly different (p<0.001).

**Table 4:** Effect of copper, zinc, and manganese supplementation on egg quality parameters during four laying periods

Items	INO	HYC-Nut	HYC-Low	HYC+INO	p-value
25 to 28 weeks					
Shell thickness (mm)	0.23	0.18	0.20	0.19	0.990
Shell weight (g)	5.14^B^	5.10B	5.26^A^	5.41A	<0.0001
Surface area (mm^2^)	73.13	72.85	73.04	73.95	0.305
SWUSA	0.070^B^	0.072^A^	0.072^A^	0.073^A^	0.0001
Broken eggs (%)	0.25	0.37	0.32	0.24	0.575
Saleable eggs (%)	99.75	99.63	99.68	99.76	0.575
29 to 32 weeks					
Shell thickness (mm)	0.19^AB^	0.18^B^	0.17^B^	0.20^A^	<0.0001
Shell weight (g)	5.32	5.33	5.18	5.28	0.7495
Surface area (mm^2^)	73.56	73.96	73.98	73.17	0.760
SWUSA	0.07	0.07	0.07	0.07	0.580
Broken eggs (%)	0.35	0.24	0.23	0.27	0.620
Saleable eggs (%)	99.65	99.76	99.77	99.73	0.620
33 to 36 weeks					
Shell Thickness (mm)	0.17	0.18	0.17	0.18	0.090
Shell weight (g)	5.02^C^	5.40^A^	5.28^B^	5.54^A^	<0.0001
Surface area (mm^2^)	74.65	75.76	75.84	75.79	0.410
SWUSA	0.069^ab^	0.071^a^	0.068^b^	0.073a	0.0100
Broken eggs (%)	0.46	0.29	0.20	0.44	0.510
Saleable eggs (%)	99.67	99.74	99.81	99.49	0.510
37 to 40 weeks					
Shell thickness (mm)	0.36	0.38	0.38	0.38	0.18
Shell weight (g)	5.52	5.74	5.64	5.50	0.08
Surface area (mm^2^)	77.92	78.09	77.67	77.72	0.480
SWUSA	0.07	0.07	0.07	0.07	0.140
Broken eggs (%)	0.45	0.37	0.31	0.39	0.700
Saleable eggs (%)	99.55	99.63	99.69	99.61	0.700

n = 100 eggs from 15 replicates (at 7 eggs/replicate in 10 replicates and 6 eggs/replicate in 5 replicates).

SWUSA, shell Weight per unit surface area.

a,b Means in a row bearing different superscripts are significantly different (p<0.05).

A–C Means in a row bearing different superscripts are significantly different (p<0.001).
